# Improving the Calibration of Image Sensors Based on IOFBs, Using Differential Gray-Code Space Encoding

**DOI:** 10.3390/s120709006

**Published:** 2012-07-02

**Authors:** Pedro R. Fernández, José Luis Lázaro Galilea, Alfredo Gardel Vicente, Ignacio Bravo Muñoz, Ángel E. Cano García, Carlos Luna Vázquez

**Affiliations:** 1 Electronics Department, University of Alcalá, Superior Polytechnic School, University Campus, Alcalá de Henares 28871, Madrid, Spain; E-Mails: lazaro@depeca.uah.es (J.L.L.G.); alfredo@depeca.uah.es (A.G.V.); ibravo@depeca.uah.es (I.B.M.); caluna@depeca.uah.es (C.L.V.); 2 Telecommunication Department, University of Oriente, Av. de las Américas, SN, Santiago de Cuba 90400, Cuba; E-Mail: acano@fie.uo.edu.cu

**Keywords:** image sensor, fiber imaging, image transmission, sensor calibration, optical fiber sensors

## Abstract

This paper presents a fast calibration method to determine the transfer function for spatial correspondences in image transmission devices with *Incoherent Optical Fiber Bundles* (IOFBs), by performing a scan of the input, using differential patterns generated from a Gray code (*Differential Gray-Code Space Encoding*, DGSE). The results demonstrate that this technique provides a noticeable reduction in processing time and better quality of the reconstructed image compared to other, previously employed techniques, such as point or fringe scanning, or even other known space encoding techniques.

## 1. Introduction

Visual inspection systems using electronic cameras can present limitations in certain scenarios where it is not possible or suitable to use electronic devices without adding a special sensor package or implementing considerable modifications to the system structure. Such scenarios include areas which are difficult to access (winding/narrow conduits), medical applications related to endoscopy and the inspection of harsh environments exposed to high temperatures, risk of explosion, corrosion, presence of radiation, *etc.*

For the transmission of images under these conditions, it is common to use coherent optical fiber bundles where the fibers retain the same spatial relationship (or position) between them. In this manner, optical fiber bundles can ensure high galvanic isolation with a flexible image acquisition structure. On the other hand, image transmission using coherent fiber bundles is only effective at close distances and entails a relatively high cost per meter of length. However, previous studies have shown that incoherent optical fiber bundles (IOFBs) may also be used for this purpose proving to be cheaper, with less inter-fiber crosstalk and capable of achieving longer working distances. From the manufacturing viewpoint, the cost of IOFBs is significantly lower because ordered distribution and fusion of the fibers are not required. The image obtained by an IOFB has an intrinsic encoding given by the random distribution of fibers. This means that for the reconstruction of IOFB images, it is necessary to perform a spatial calibration of the system for estimating the transfer function (typical of each bundle) for recovering or decoding the information captured by the sensor [1–4].

**Figure 1.**
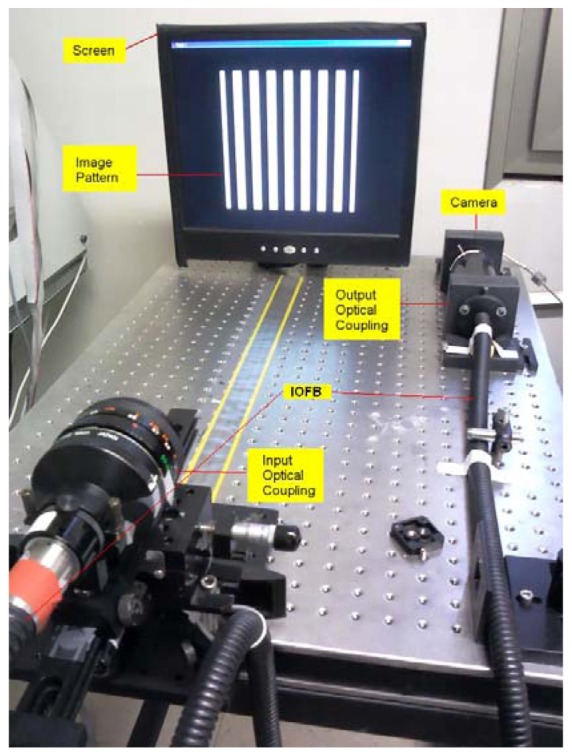
Structure of a typical calibration/transmission IOFB system.

A typical calibration/transmission IOFB system is structured as shown in Figure 1 [1–4]. All control of both the sensor and calibration screen is performed from centralized processing hardware that is also involved in the capture process and formation of the final image. The system requires a sensor or adapted camera coupled to a processing unit that reconstructs the information received at the output of the fiber bundle.

IOFB calibration not only entails geometrical calibration, but also implies parameterization of the response to light of the fibers because this is affected by the transfer functions of each fiber (attenuation) and their optical coupling. Given a homogeneous input of light to the IOFB, the parameterization will provide a uniform gray level for all pixels in the image [5].

In [4], it was shown that the use of spatial encoding techniques provides greater computational speed during the calibration procedure. However, although they are valid, they do not surpass the quality and accuracy given by the Fringes Method described in [3]. In this method, the patterns consist of bright regions or fringes of variable width that are encoded using a predetermined function [1,4]. This paper presents a new calibration method that scans the spatial optical input of the bundle using image patterns that are projected from a monitor screen onto the entrance of the IOFB via an optical coupler, and the transfer function of the system is determined by sequentially verifying the response of each IOFB fiber for the whole set of images captured by the sensor. The calibration process gives a look-up table denoted as the Reconstruction Table (RT). This table is unique to each IOFB and has a fixed format, considering as many records as there are fibers in the image sensor.

### Preliminary Work Issues Related to Spatial Calibration

Previous studies have reported the requirement to locate the fibers prior to spatial calibration in order to know in advance where to extract useful information from each image taken by the sensor coupled to the IOFB. The location of fibers can be obtained by a detection algorithm for circular patterns considering a single radius. This problem was easily solved in [5,6], and has a direct relationship to the procedures of image formation and correction regardless of the sweeping method used.

In [7], a methodology was presented for focusing the optical input and some simple metrics were proposed in order to correct blurring. The authors also indicated that the degree of focus in images projected onto the entrance, the resolution of the optics used in the input subsystem and lens aberrations could produce a blurring effect on images that would impact on the input and thus decisively affect the quality of the calibration since it causes a dispersion of the incident energy. For these reasons, this focusing technique will be used as part of our proposal.

Several calibration techniques have been reported in the literature. The main difference among them is the type of light scanning used, but they have several intermediate steps in common and, in many cases, the optical setup. Scanning can be performed of square regions, fringes, or light beams using spatial coding techniques generally based on binary codes [1–4].

In [4], a calibration method was reported for spatial encoding based on a *Differential Binary Spatial Encoding* (DBSE), using a reduced set of differential images (paired) and a correction procedure for the results which enhances the quality of the calibration and reconstructed images. These patterns are generated according to a weighted binary code and it is an efficient way to scan the input bundle because the number of images to use is considerably reduced, as is memory use. When the behavior of each fiber at the output for each pattern image is known, the corresponding positions at the entrance can be calculated. The results of this analysis of all the pattern images form the RT. The importance of this method is that the input-output relationship is obtained with a significant reduction in the number of the images required for decoding the system compared to the method described in [2], and therefore, less time is required for analysis and memory requirements are lower as regards 2D scanning techniques using square regions, fringes or space encoding. Furthermore, the use of differential images facilitates and ensures better decoding results compared to the results obtained with [2].

The use of differential images provides greater certainty about the state of a particular fiber for each pattern. Furthermore, this paper introduces a method for analyzing the initial results of the RT to correct redundant data and discard outliers. This RT optimization gives a better quality of calibration and enhances final images. However, although DBSE can achieve an outstanding calibration, it is very sensitive to the degree of image focusing and the intrinsic quality of the input optical subsystem. To minimize this problem, image patterns are subdivided into images with fewer lighting changes.

The present study describes a new proposal for IOFB calibration which is based on image pattern scanning using Differential Gray-Code Spatial Encoding (DGSE), in order to improve on the results obtained with DBSE while maintaining its best features, and to achieve a fast and efficient method providing high quality final images. In Section 2 below, some preliminary considerations in IOFB calibration are discussed.

## 2. Image Scanning and Reconstruction Table Models. Primitive Image

The scanning space for the system input may be seen as an imaginary grid composed of uniformly sized square cells with a surface area similar to the one occupied by an IOFB standard fiber. Thus, each side (*l*) of a square cell has a length equivalent to the average diameter of IOFB fibers *d_fib_* (3/4*d_fib_* ≤ *l* ≤*d_fib_*). Each image pattern is completely projected from the calibration screen onto this input plane. Each square cell may be seen as an area where each image pattern impacts with a lighting value, forming a unique sequence of changes in intensity for the whole set of patterns projected, which makes it distinguishable from any other cell in this 2D scanning range [4]. Thus, the algorithm should find the output fiber which follows a predefined sequence of changes in the corresponding input, considering that the whole set of patterns is projected sequentially. Then, the geometrical position of an input cell will be paired with a fiber position in the output image captured by the sensor. This pixel coordinate corresponds to the fiber that best follows the changes of light generated in a given input cell. It also implies that for each pattern image, two cell position codes should be obtained referring to a pair of horizontal and vertical coordinates.

Given this association model (“one fiber–one cell”), the RT will have a total number of records which depends on the number of detectable fibers in the image captured by the sensor. Each row in the RT represents a localized fiber centroid that is associated with a position in the input cell as well as an equalization factor that corrects attenuation in each fiber. The centroid *i* of each fiber refers to the 2D coordinate system of the camera (r(*i*), c(*i*)). The corresponding input cell represents the discrete position (R(i), C(i)) to which the information extracted from the central region of a determined fiber should be transferred. Thus, the general structure for the RT shown in Table 1 is similar to those given in [3–6].

The first two elements of the RT, r(*i*) and c(*i*), are obtained by the FDDT (*Fiber Detection using Distance Transform*) method for locating fibers given in [5,6]. The FDDT method requires that the calibration screen project a bright, uniformly illuminated image without presence of saturation effects in the image output. The results of this scanning are the first data to be included in the RT, together with the equalization factor α(*i*). In order to clarify the contribution of our paper, a single equalization factor will be considered for grayscale images. To calculate the equalization parameter, the simple and fast method described in [3] is applied. Values R(*i*) and C(*i*) are obtained from subsequent processing of all images captured by the sensor during the scanning procedure. The status of all fibers in each image pattern captured by the camera must be verified and analyzed following the order of appearance during the sequential sweep. The generated image patterns are composed of multiple black and white fringes. The status of each fiber against a specific image pattern is determined by taking into account the behavior for both differential images.

**Figure 2.**
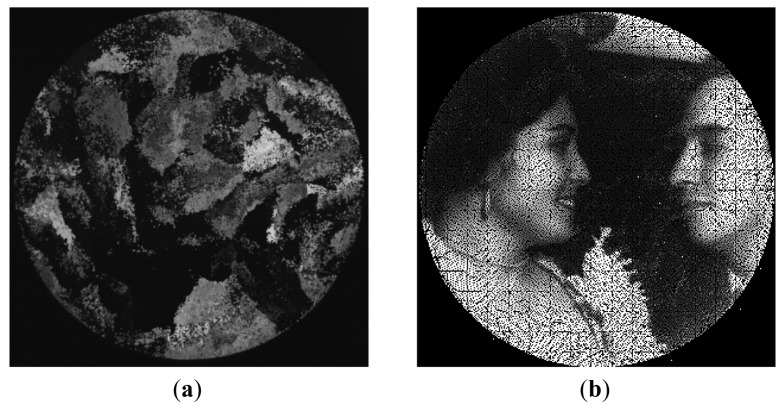
An example of an image captured by the sensor (**a**) cluttered image *Id* (**b**) primitive image *Ip* obtained through RT application.

The distribution of the fibers of an IOFB is unknown, thus the dimensions of scanning space should be estimated according to the maximum theoretical number of cells that might exist. In order to determine this theoretical maximum number of fibers (*nfib*_max_), it is assumed that all fibers are perfectly aligned in a square grid and have a uniform diameter. This value also determines the maximum number of cells (*n_celd_*) for each side of the square, giving a final image size of *n_celd_* × *n_celd_*. To determine the integer value of *n_celd_*, the following equation is used:

(1) $$n_{\text{celd}}=\text{nfi}b_{\max}\approx \frac{B_{\text{diam}}}{F_{\text{diam}}}$$

where *B_diam_* is the IOFB diameter and *F_diam_* is the nominal fiber diameter. A fiber will be considered as illuminated when at least more than 50% of its area is covered by one white stripe from the image pattern projected on the IOFB. This percentage ensures that the response of any illuminated fiber can be distinguished with respect to an unlit state. The primitive image (*Ip*) (Figure 2) is obtained by applying the RT table and reordering the information contributed by the fiber illumination.

This primitive image presents a certain number of empty regions which correspond to regions without fibers in the input of the IOFB, due to interstitial gaps and also possible calibration errors in fiber location.

From [3–6], it is known that the *Ip* image has a size of *n_celd_* × *n_celd_* points, and three different types of regions can be extracted. The first type of region corresponds to the set of pixels *E* located outside the contour of the bundle (a circular IOFB in our case), with an intensity value of 0. The second type of region corresponds to the set of locations *I* where the RT table has given an illuminated value. And finally, the third region consists of those interstitial gaps where the intensity values are not yet known. According to the above classification of regions, *Ip* image values may be obtained using the following equation:

(2) $$\begin{array}{l} Ip(R(i),C(i))=\text{med}(Id(N_{9}\{r(i),c(i)\}))\cdot \alpha (i)\\ \forall i=1\ldots n_{\text{celd}}\in I \end{array}$$

From the cluttered image (*Id*), the median of the neighborhood intensities contained in a 3 × 3 pixel window (neighborhood N_9_) is calculated for each fiber position, and that value is multiplied by the equalization factor α*_i_* which has been previously calculated and stored in the RT. This result is transferred to the input grid position *Ip*(R(i), C(i)) as stored on the RT. The remaining positions (*E* ∪ *U*) give the following values:

(3) $$Ip(R^{\prime },C^{\prime })=\{\begin{array}{l} 0\to \forall ({\text{R}}^{\prime },{\text{C}}^{\prime })\in E\\ \text{NaN}\to \forall ({\text{R}}^{\prime },{\text{C}}^{\prime })\in U \end{array}$$

where (R′, C′) represent the *Ip* coordinates that are not included in the RT. The *NaN* (not-a-number) may help to identify unknown regions for future image *inpainting* procedures [8].

## 3. Experimental Section

### 3.1. Decoding the DGSE Technique

This paper proposes a calibration methodology to obtain the RT using an image scanning method based on Gray code. The method emerges as an alternative to other spatial encoding methods for minimizing errors that can occur when estimating the state of fibers during the IOFB input scan. These errors have a direct relationship to the degree of focus and resolution of the optical subsystem. In this sense, any blurring effect caused by the degradation of these factors will produce energy dispersion in the captured images and impact negatively on both the quality of the calibration and the final image result [4]. DGSE establishes differential processing of the captured images without an excessive increase in the number of images necessary. Differential processing of image patterns involves the generation of another, complementary image for each base pattern image.

**Figure 3.**
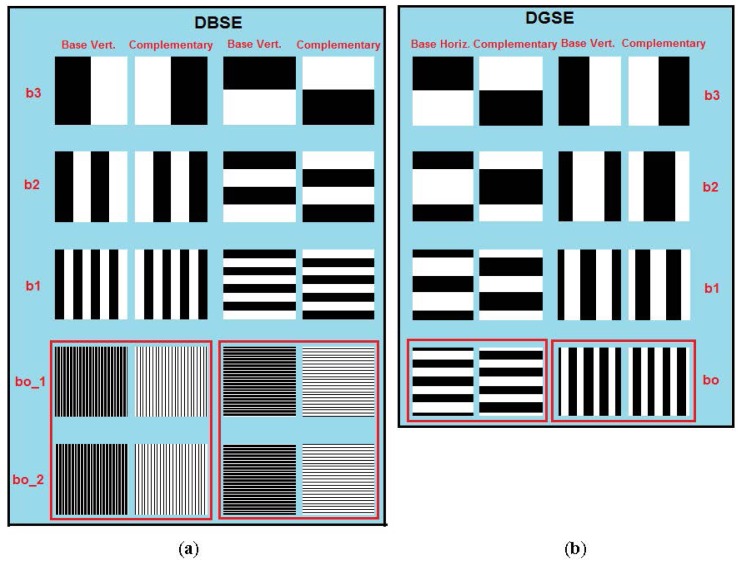
(**a**) DBSE and (**b**) DGSE scanning image patterns with a number of bits (n_bits_ = 4 bits). Note the lower spatial frequency of DGSE with respect to DBSE.

It is well known that the Gray code is a binary, non-weighted numerical system where two consecutive values only differ by one bit. This feature makes it relatively easy to minimize the occurrence of duplicated records in the RT because transitions from 1 to 0 and 0 to 1 (especially in the least significant bit) occur with a lower frequency than that experienced with the weighted binary code used in DBSE. It also avoids the need to subdivide the images corresponding to the least significant digit in order to better differentiate the states of the fibers [4]. Figure 3 shows the images necessary, considering a scanning space of 16 × 16 cells (n_bits_ = 4 bits) for comparison of the DBSE and DGSE methods. As in DBSE, differential processing should be applied to the captured images during the scanning task. For this reason, the analysis of the images to obtain the RT will be similar to [4], but will consider the different encoding method used.

The use of differential images is based on the principle that a fiber is considered optimally illuminated by a base image pattern if it is unlit when its corresponding complementary image pattern is projected. If an excited fiber remains slightly illuminated for both differential patterns, it is considered unlit, because it has not undergone a significant change in its status and the condition analyzed is not conclusive. In this case, the fiber is considered neither illuminated optimally nor sufficiently unlit (remaining in an undetermined state), and for this reason, it should be considered unlit.

Taking a scanning space of 256 × 256 cells as an example, the DBSE method analyzed [4] required about 36 images for both dimensions. Under the same physical conditions, the DGSE method needs to generate only 32 images, including 16 differential pairs for each dimension (16 + 16 = 32 images). The number of images is much lower than that required to scan the IOFB using the Fringes Method (see [3]), which would require 512 images (2 × 256) with high resolution to ensure proper decoding.

Described below are the procedures for calculating the RT parameters. Note that in order to determine the status of each fiber during scanning, it is essential to know in advance the centroids of the fibers and the equalization factors *α_i_*. The procedure used to determine these initial values was the FDDT technique described in [5,6], assuming that all the fibers have a similar diameter (nominal diameter).

### 3.2. RT Generation

Below, the necessary procedures proposed for calculating the rest of the parameters included in the RT are described. The RT generation process is similar to the method described in [4], maintaining the same RT structure. The captured images are initially loaded into memory during the scanning process, preserving the sequence of appearance, and a subtraction is performed between each differential pair of images, according to the following operation:

(4) $$I_{Rn}=\left\{\begin{array}{c} I_{Pn}-I_{Pn_{D}},I_{Pn}>I_{\text{PnD}}\\ O,I_{Pn}<I_{\text{PnD}} \end{array}\right\}$$

where I_Rn_ is the image resulting from the subtraction of the complementary images with subscript “*n*” captured by the camera. The next step is to check the status of each fiber (lit or unlit) for each pair of differential images for a given orientation (vertical or horizontal) of the scanning range. The subtraction operation enables us to reject the fibers that, in response to a differential pair of images, present an “indeterminate” response because they are physically located in the middle of a light-dark transition border or *vice versa*. According to the sequence of states that occur in the fibers during the scan, a pair of “position codes” (row and column) are generated and stored in memory. From these codes, an approximate cell location is determined and stored in the RT by converting these Gray codes into numeric values. Each position code bit is associated with an image pattern number and its value will be related to the state of the fiber at that sweeping instant.

To determine the fiber status in each *I_Rn_*, the median gray level *I_MED_* around the centroid (*u_ci_*, *v_ci_*) of the fiber analyzed is calculated, taking into account a neighborhood window of nine pixels (N_9_ → 3 × 3 pixels) such that:

(5) $$\{\begin{array}{c} I_{\text{MED}}\left(N_{9}\left(u_{c_{i}},v_{c_{i}}\right)\right)=\text{Med}\left(I_{Rn}\left(u,v\right)\right)\\ \forall \left(u,v\right):\left\{u_{c_{i}}-1\leq u\leq u_{c_{i}}+1,v_{c_{i}}-1\leq v\leq v_{c_{i}}+1\right\} \end{array}$$

If the median gray level exceeded a threshold value *δ_GL_*, the fiber is considered “lit” and is associated with the logical value “1” in the bit position code corresponding to the image pattern. The position of the corrected bit will also correspond to the order of appearance of the image analyzed. If it did not exceed the threshold mentioned, the fiber is considered “unlit” and is associated with the logical value “0” in the bit corresponding to the position code, as expressed in the following formula:

(6) $$\{\begin{array}{c} I_{\text{MED}}>\delta_{GL}\to "1"\to "\text{lit}"\\ I_{\text{MED}}<\delta_{GL}\to "0"\to "\text{unlit}" \end{array}$$

Due to the differential processing of the images involved in this analysis, the *δ_GL_* values should be very close to 0, as shown in [4]. An Intensity value *I_MED_* > 0 indicates that a fringe is covering the fiber core, at least, a 50% of his area. In that case, the fiber is receiving enough energy and can be consider it as lit. After completing this analysis, the final result is a preliminary RT generated in a very short time, even less than with DBSE, since it analyzes a smaller number of images. Note that the number of images to be stored for later analysis can be reduced by half due to the implicit subtraction operation described in Equation (4).

### 3.3. RT Refining

Once the preliminary RT has been obtained, it must be optimized to verify the possible existence of records that are considered outliers, duplicates or simply empty. The method followed is similar to that used in [4], but employs encoding based on a Gray code. Each pixel that makes up the original image must fulfill a physical model that is consistent with reality. Thus, no fiber must be found outside the physical limit imposed by the circular shape of the bundle, nor can cells be associated with more than one fiber position. By ensuring a suitable focus and a correct projection of the image onto the bundle, the number of outliers remains low. Any entry that does not fulfill this physical model should be corrected or removed from the preliminary RT.

After this first step in RT optimization, the analysis continues to identify those groups of cells whose positions are redundant. These records violate the correspondence between fiber and cell position under the system model. In each group, the best matching record should be detected. To simplify the process, the RT is ordered by cell positions. Thus, those redundant records that dispute over the same cell will be grouped consecutively, facilitating their identification and correction.

Each group of records is analyzed separately, looking for the “best entry” of a current cell position. The “best entry” from a group disputing over the same cell is the one closest to an ideal condition (described below) and it will remain unchanged in the RT. The rest should be relocated to neighboring empty cells (if any) that were not considered in the RT. If a record cannot be reallocated, it is removed from the RT.

We considered that ideal fiber excitation (or an ideal condition) existed when each time the fiber was illuminated from the input, it attained its maximum level of light transfer and, on the other hand, when it was unlit it reached the minimum degree of intensity at the output. If these ideal conditions always occurred in the fiber, this would indicate that each fringe excitation produced the maximum superimposition on its nucleus at the input, and, in contrast, when it was unlit it would indicate that it was not receiving any influence.

Normally, this does not always occur; a fiber is more or less illuminated depending on the degree of the fringe superimposition on its nucleus. However, bearing in mind the sequence of gray levels that should be obtained under ideal conditions and comparing this with the real sequence, an idea is obtained of the extent to which the result obtained resembles that sought. In other words, the ideal condition serves as a reference for comparing the different entries of a group in dispute and defining which is the best candidate for that cell.

When the maximum gray level $\left(g_{\text{max}}^{i}\right)$ reached by each fiber during the scan is known, a pattern values vector (or pattern chain) can be constructed from *n* bits by means of:

(7) $$Cp\to \left[b_{n-1}\cdot gi_{\max},\ldots \ldots b_{2}\cdot gi_{\max},b_{1}\cdot gi_{\max},b_{0}\cdot gi_{\max}\right]$$

where *b_k_* is the weight {∀*k* = 0, 1, …, *n*} which has the value of 1 for the “illuminated” fiber and 0 for the “unlit” fiber, and $g_{\text{max}}^{i}$ is the maximum level of gray that has been registered for the fiber *i* by the sensor. This representation is analogous for rows and columns, and thus each fiber will have its own pair of ideal sequences. Similarly, considering ${\text{g}}_{k}^{i}$ as the average real level of gray reached by the fiber *i* in the image *p* = {0, 1, 2, n − 1}, then, for each redundant entry we obtain the vector:

(8) $$Cr\to \left[b_{n-1}\cdot gi_{n-1},\ldots \ldots b_{2}\cdot gi_{2},b_{1}\cdot gi_{1},b_{0}\cdot gi_{0}\right]$$

To analyze the degree of similarity between the pattern and redundant chains, the following metric based on the root mean square error is used:

(9) $$\text{RMSE}=\sqrt{\frac{\overset{p=n-1}{\underset{p=0}{\text{∑}}}{\left(b_{p}gi_{\max}-b_{p}g_{p}\right)}^{2}}{n}}$$

The combination giving the least error out of the redundant cell combinations (best entry) is chosen and remains in the RT. The rest of the redundant entries are relocated toward the positions of neighboring cells not registered in the RT, where errors between pattern chains *Cp* (row and column) and *Cr* are also minimized. These values are temporarily stored and verified again to check whether new redundancies appear when all entries are verified again. If any cases remain unresolved after a specific number of iterations, these are definitively eliminated from the RT since they may be associated with false fiber detection, and where they arise, their number is very low compared to the remainder of validated entries. It is worth noting that the number of records removed is very low compared to the number of records validated, as will be discussed in the results section.

### 3.4. Experimental Setup

**Figure 4.**
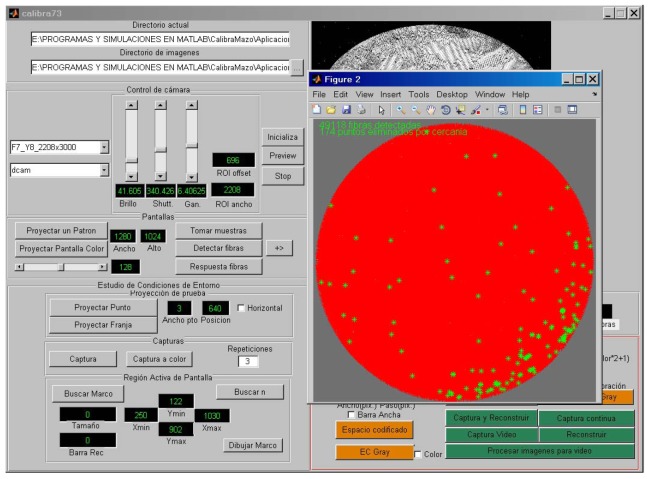
Graphical interface for calibration.

The results presented in this paper were obtained with the same instrumentation and laboratory equipment as used in [4] (see Figure 1). The instrumentation is controlled through a Matlab graphical interface containing all the operations necessary to perform the spatial calibration of the system and to assess multiple calibration methods (Figure 4). All algorithms were implemented on a PC based on a 3 Ghz Pentium Core 2 Duo with 4 GB of RAM.

The camera used to capture images through the IOFB was a monochrome C-CAM camera BCi4-6600 with a 6.6 Megapixel CMOS sensor and 2,208 × 3,000 pixels of resolution coupled to the bundle through a lens specifically designed to ensure that the image of the bundle matched the sensor size in the smallest dimension (vertical). The camera resolution was chosen assuming that each fiber has an effective area of about 7 × 7 pixels in order to ensure an adequate location of the fibers in the output image through the FDDT method.

The input of the IOFB was coupled with a 19–35 mm optical zoom. Both the sensor and screen were isolated by a black box (not shown in Figure 4), to avoid the influence of any external sunlight, artificial illumination changes and reflections on the calibration screen. The calibration screen was a typical 17″ desktop monitor (AOC LM725) with 1,280 × 1,024 pixels, a pitch of 2.64 μm, and brightness up to 250 cd/m^2^. All experiments were performed with a 2.8 m long fiber bundle containing about 50,000 fibers with a nominal diameter of 50 microns [9,10]. Given these characteristics, *nfib*_max_ is approximately 256 fibers in both dimensions.

**Figure 5.**
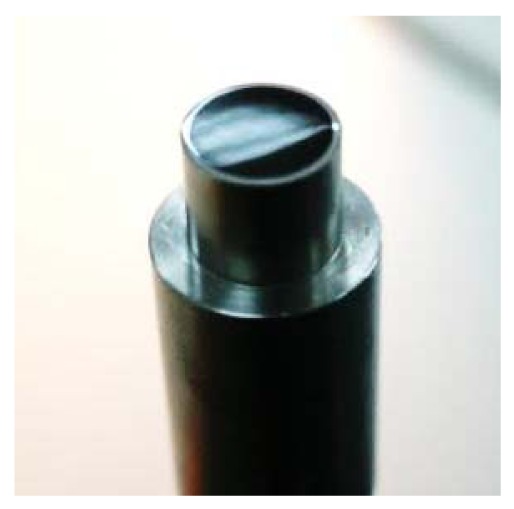
Polished IOFB containing approximately 50,000 fibers.

To improve the light transfer and remove some epoxy blobs on the surface of the IOFB, both sides (input/output) were polished (Figure 5). According to the installation geometry, an active area on the calibration screen of 768 × 768 pixels was chosen empirically.

### 3.5. General Calibration Procedure for DBSE and DGSE

**Figure 6.**
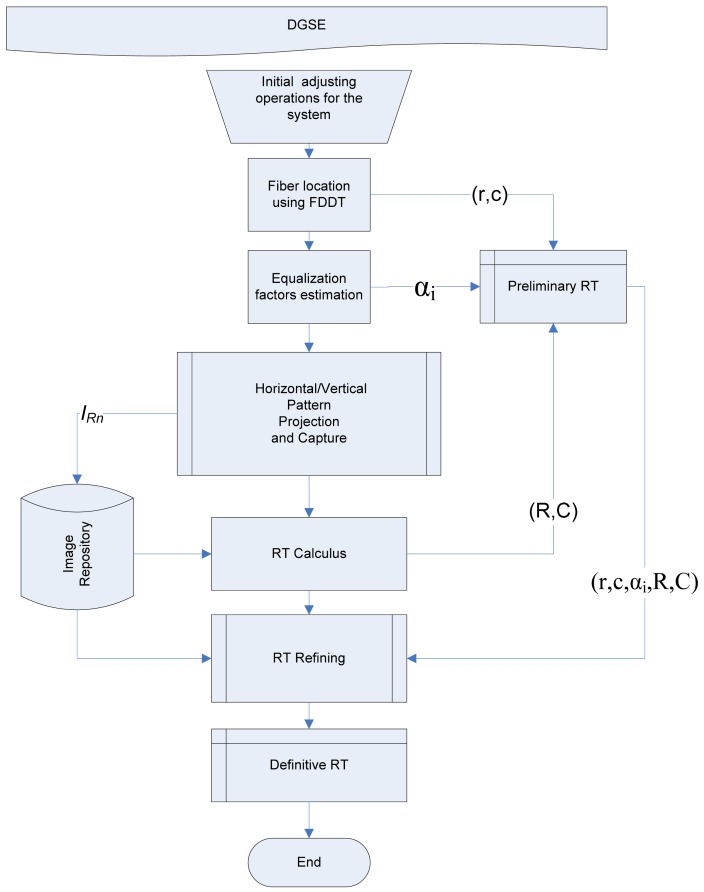
General procedure for DGSE.

This study inherits from [3–5,7], respectively, the methods for fast detection of fibers (FDDT), focusing based on the *f_var_* metric and scanning methods based on Fringes and DBSE techniques. The procedures described below are common to both DBSE and DGSE techniques and must be carried out in the order given (see Figure 6):

1. Focus the optical input of the bundle using the *f_var_* metric given in [7] and adjust the position of the input of the bundle so that it completely captures the active area of pattern images in order to optimize the scanning space.
2. Locate all the fiber positions in an image captured by the camera. This is carried out by means of a FDDT algorithm applied onto an image of the bundle illuminated homogeneously, which enables rapid location of the fiber centroids.
3. Calculate the equalization factors αi which will compensate the response of the fibers. These equalization parameters are also entered into the preliminary RT.
4. Once the entire system has been adjusted, perform sequential scanning of the IOFB input with the projection of differential image patterns, correctly synchronized with image shutter acquisition from the camera. The images I_Rn_ are stored for further analysis in files named according to their numerical position in the Gray encoding used while sweeping along each dimension.
5. For each resultant image *I_Rn_*, the fibers previously located using FDDT and showing high levels of excitation will be stored in a table. This operation makes it possible to generate a binary position code for each fiber position along horizontal and vertical dimensions. These Gray codes are converted to corresponding binary numbers and stored in the preliminary RT.
6. Once the preliminary RT has been generated, RT refinement is carried out by removing the outliers and the redundant coordinates. Once the system has been calibrated and the RT refined, it is necessary to verify that calibration is correct.

## 4. Results and Discussion

Table 2 shows a comparison of different calibration methods comprising scanning methods such as Fringes, DBSE and DGSE. These results were obtained under the same working conditions: camera configuration, optical couplings, hardware, controlled external lighting, *etc.* The comparison reveals that the Fringes Method is the most time consuming during scanning and processing tasks, as well as requiring most memory. This is mainly due to the need to process a greater number of high-resolution images than with DBSE and DGSE. Despite these drawbacks, the technique achieves very good results, and a high calibration quality with few records to be corrected in the preliminary RT compared to DBSE.

In the Fringes method, the maximum error calculated for any cell position is in the range of ±1 cell position along each dimension, because the patterns are not generated using a base code. In contrast, in the DBSE method, if an error occurs in the status estimation of a fiber during scanning, it could generate a large error in the position code of the fiber, depending on the weight of the affected bit.

Methods based on spatial encoding quantitatively outperform the fringes technique in terms of fast processing speed and low memory requirements. However, it is known from [4] that DBSE is very sensitive to both focus and optical input resolution. This is because the spatial frequency of the fringes increases gradually during the sweeping. The results presented in Table 2 show that the number of records removed from the RT reaches 13%. This value is higher than with the Fringes or DGSE methods (5.4% and 4.65% respectively).

The DGSE method is an evolution of the DBSE technique, exploiting the same differential processing of the captured images while reducing its weaknesses. Although RT generation speed is not the most critical factor in the calibration process, rapid generation is nevertheless a desirable feature. In this sense, the DGSE method obtains the best scanning and processing times. However, its most remarkable feature is that it provides the largest number of valid records in the final RT, over 95% of initial entries. Another advantage that is inherited from DBSE is that the optimal threshold in DGSE, used as a reference for the current state of the fiber, has a value close to zero, thus avoiding the need for any optimization process. This is a disadvantage present in the Fringes Method, where this threshold must be calculated using an iterative optimization procedure which increases the calibration time.

To obtain results similar to those of DGSE with Dujon or DBSE techniques [1,4], it would be necessary to use higher resolution optics or a larger scanning space. Otherwise, the number of outliers and errors in the estimated position codes could rise significantly. Increasing the resolution of the spatial scanning has the disadvantage of causing greater dispersion of the pixels in the final image. For example, one extra bit in the base code used in image patterns increases the scanning space from 256 × 256 to 512 × 512 cells, which in turn would increase the sizes of the interstitial regions, which is not suitable for the reconstruction of the final image.

**Figure 7.**
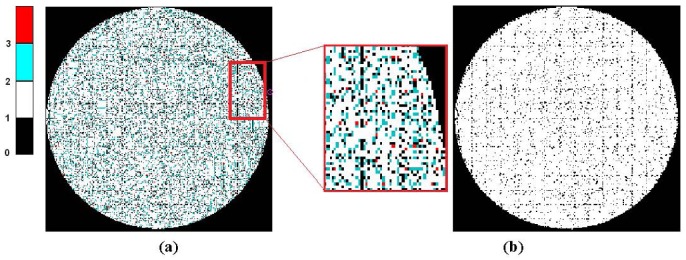
DGSE RAMs. (**a**) RAM for the initial RT; (**b**) Final RAM (binary values).

To focus the analysis on the effect of redundancies correction on the RT, a *Redundancies Accumulation Mapping* (RAM) will be used. Figure 7(a) shows the RAM for the initial RT (with a larger number of redundancies). RAM values accumulate the number of assignments for any particular pixel which can be related to one or more records from the preliminary RT. The value of each pixel corresponds to the number of occurrences for that coordinate cell in the RT. Figure 7(b) shows the final mapping without any redundancies, optimized by the DGSE method.

If the RT has no redundancies, then the RAM image is binary and is equivalent to an original image of a white projected image (Figure 7(b)). It can be seen that within the outline of the bundle, a number of small interstitial regions remain. These uncertainties in the reconstructed image are mainly due to irregular distribution of the fibers and the quality of bundle packing. These regions will be reconstructed using the information in neighboring pixels in order to enhance the quality of the final image.

**Figure 8.**
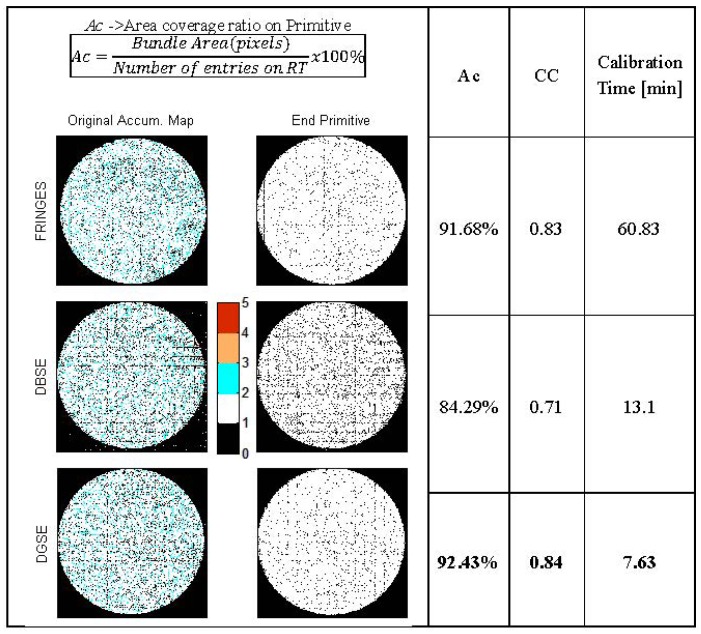
RAMs and Primitive Images for different methods.

Figure 8 shows the evolution of iterative RAMs when applying the correction of redundancies to the methods discussed in Table 2. From Table 2, it can be seen that the DBSE method has more redundancies and leaves more interstitial gaps, whereas the DGSE results are slightly better than the Fringes method. A coverage ratio of the original image area (Ac) has been estimated, indicating how efficiently the primitive image covers the contour of the bundle. The figure also presents the similarity between the primitive and the bundle contour (white circle) through a correlation coefficient (CC) which shows that the DGSE and Fringes methods provide the best results, far exceeding those of the DBSE method.

The *inpainting* algorithm for undetermined regions is an essential procedure in order to achieve an adequate reconstruction of the final image which is consistent with its original structure. For this purpose, different techniques are commonly used based on variational calculus, partial differential equations, convolution masks, *etc.* Although it was not the aim of this study to make a contribution in this area, it is interesting to show the general characteristics of undetermined regions to be regenerated. To understand the problem, let us define the Primitive Neighborhood Maps (PNM). These maps have the same size as the primitive image and represent, for each undetermined pixel labeled with a *NaN* value (see the Equations (2) and (3)), the number of neighboring pixels that are known. All known pixels have a neighborhood value equal to 0 (N_0_), to be differentiated from the undetermined pixels. The rest of the pixels have a value between 1 and 8 (N_1_, N_2_, …, N_8_), according to the number of known neighboring pixels.

**Figure 9.**
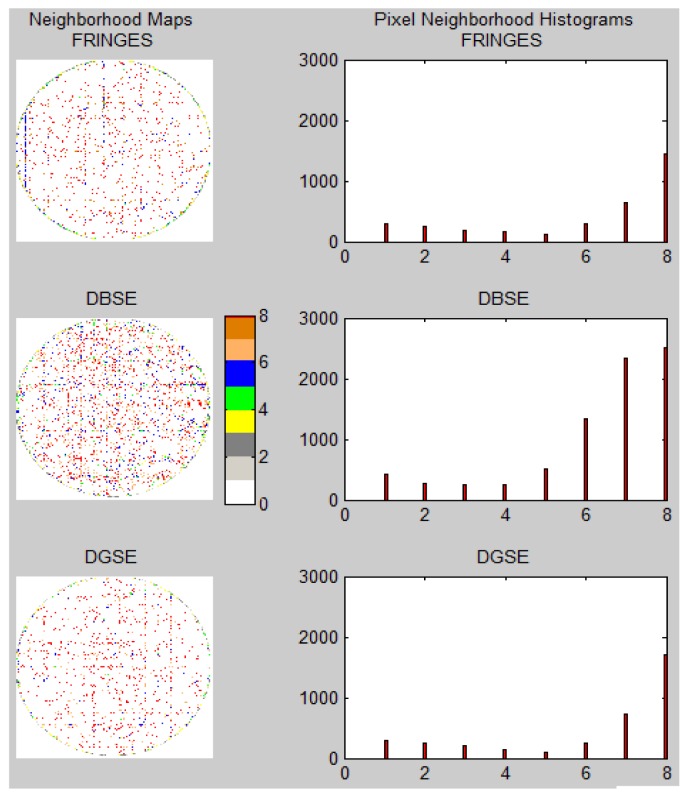
PNMs and histograms for the calibration methods discussed in Figure 8.

Figure 9 shows the PNMs for the examples given in Figure 8, as well as the corresponding neighborhood histograms. It can be seen that the DBSE method has more unknown positions than the DGSE and Fringes methods. In the histograms, N_0_ values were omitted from the accumulation register in order to focus attention on the unknown positions and their characteristics.

The histograms show that most of the unknown pixel bins are concentrated in N_8_ and N_6_. This fact could facilitate the *inpainting* process due to the high amount of information available around many unknown cells, and therefore an *inpainting* technique based on masks, such as the one from Oliveira in [8], could be applied.

**Figure 10.**
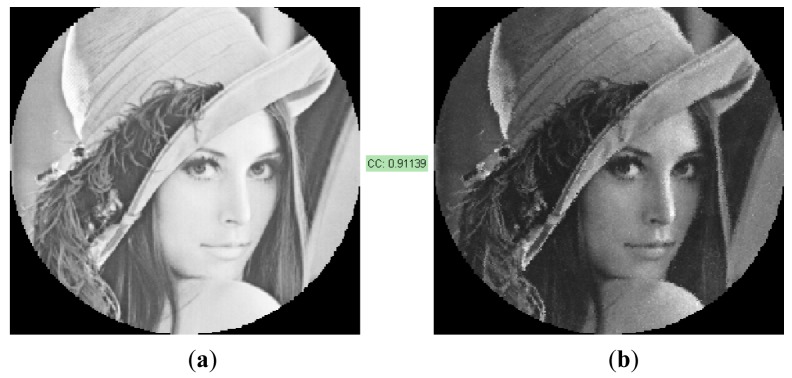
Image reconstruction and correlation result. (**a**) Original image (**b**) Inpainted image using the Oliveira method.

Figure 10(b) shows an example where a primitive image has been fully reconstructed using the fast *inpainting* technique of Oliveira designed to reconstruct small unknown regions. It can be seen that the images have a large contrast ratio, and current spatial resolution is 254 × 254 pixels according to the IOFB system under test. The level of similarity expressed through the correlation coefficient is high (0.911).

Comparing the results of this example with those shown in [4] for the same image, it can be seen that although DGSE performs better than DBSE, the results of the reconstructed images are very similar (0.911 *vs.* 0.909 respectively). This is because of the improvement achieved in the end image by means of the *inpainting* procedure.

## 5. Conclusions

This paper presents a new technique for the calibration of image transmission systems based on IOFBs, and demonstrates that these systems are a viable alternative to those based on coherent fiber bundles. The DGSE method presented is based on a spatial encoding technique using differential fringe patterns generated from a Gray code.

For benchmark comparison, the scanning techniques based on the Fringes and DBSE methods were used to validate the calibration model proposed. Experiments show that all the proposed methodology is valid and capable of providing good image transmission results, although it is necessary to highlight the need to ensure that certain conditions are met:

- Good fiber location. This issue was solved efficiently by the FDDT technique described in [5,6].
- Correct focus of the optical input, which can be solved using the *f_va_*_r_ metric described in [7].

From the results of the experiments presented in this paper, the following can be concluded:

- The DGSE method is a valid proposal because it achieves good image quality and performs better in all aspects compared to the DBSE and Fringes methods. It also achieves better results in terms of the number of generated outliers and redundant entries.
- The results given in Table 2 show a validation for DGSE of over 95% of the RT records considering the initial number of records obtained by FDDT. This ensures the best quality in the formation of primitive and final images of all the methods discussed. Less than 4.5% of the initial information is discarded.
- The redundant coordinate correction procedure permits most of the ambiguous cases to be reordered to other optimal positions, providing an outstanding improvement in the active pixel area of the formed image.
- From the techniques referenced in this paper, the DBSE technique presents the worst results in terms of reconstruction quality, discrimination of the state of the fibers and outlier generation. It also has a tendency to generate large errors in the event of poor focus and requires better quality optics.
- The Fringes method is comparable to DGSE as regards the number of validated records: therefore, both methods guarantee a very similar quality of calibration. However, the Fringes method is inferior to DGSE in terms of processing speed, memory requirements and storage operations.
- DGSE recovers changes in the state (lit/unlit) of fibers during the sweep more efficiently. Thus, the threshold level used is zero, thereby rendering it unnecessary to optimize this parameter.

Once the system has been calibrated, future work will address how to use the RT with a lower resolution sensor system (at least *nfib*_max_ × *nfib*_max_ pixels), implementation on embedded hardware for image transmission and definition of a faster *inpainting* algorithm to reconstruct small areas of the image.

## Figures and Tables

**Table 1.** RT structure.

**r(i)**	**c(i)**	**α_i_**	**R(i)**	**C(i)**
where:				
(r(*i*), c(*i*))→	Coordinate pairs of the fibers located in the sensor.			
α*_i_*→	Equalization factors due to attenuation.			
(R(*i*), C(*i*))→	Position of the cell that excites the fiber most *(r(i)*, *c(i))*.			

**Table 2.** Comparison between different calibration techniques analyzed.

**Parameters**	**Method**		

	**Fringes**	**DBSE (8 bits)**	**DGSE (8 bits)**

Number of fibers located. Initial RT entries	49,127	49,127	49,127
Final validated entries	46,454 (94.5%)	42,711 (86.9%)	46,839 (95.3%)
Corrected entries (redundant)	3,270	5,867	3,799
Removed entries	2,672 (5.4%)	6,416 (13%)	2,288 (4.65%)
Mean scantime	7.91 min	5.54 min	4.92 min
Mean RT calculation time	38.94 min	2.20 min	2.08 min
Mean analysis time of redundancies and outliers	13.98 min	5.36 min	0.63 min
Number of images used/stored	522	36/18	32/16
Final image size [pixels]	261 × 261 *	254 × 254	254 × 254

* A scan space of 261 × 261 images in each dimension was considered. This inflated size of the grid is subsequently corrected in the RT so that the size of the image is not greater than *nfib*_max_ = 256 in each dimension, eliminating those cell positions that do not have an appreciable influence on the fibers.
